# Calcium‐channel blockers: Clinical outcome associations with reported pharmacogenetics variants in 32 000 patients

**DOI:** 10.1111/bcp.15541

**Published:** 2022-10-06

**Authors:** Deniz Türkmen, Jane A. H. Masoli, João Delgado, Chia‐Ling Kuo, Jack Bowden, David Melzer, Luke C. Pilling

**Affiliations:** ^1^ Epidemiology and Public Health Group, College of Medicine and Health University of Exeter Exeter UK; ^2^ Department of Healthcare for Older People Royal Devon and Exeter Hospital Exeter UK; ^3^ UConn Center on Aging University of Connecticut Farmington Connecticut USA; ^4^ Connecticut Convergence Institute for Translation in Regenerative Engineering University of Connecticut Farmington Connecticut USA; ^5^ Exeter Diabetes Group (ExCEED), College of Medicine and Health University of Exeter Exeter UK

**Keywords:** calcium‐channel blockers, epidemiology, genetics, hypertension, pharmacogenomics

## Abstract

**Aims:**

Pharmacogenetic variants impact dihydropyridine calcium‐channel blockers (dCCBs; e.g., amlodipine) treatment efficacy, yet evidence on clinical outcomes in routine primary care is limited. Reported associations in pharmacogenomics knowledge base PharmGKB have weak supporting evidence. We aimed to estimate associations between reported pharmacogenetic variants and incident adverse events in a community‐based cohort prescribed dCCB.

**Methods:**

We analysed up to 32 360 UK Biobank participants prescribed dCCB in primary care (from UK general practices, 1990–2017). We investigated 23 genetic variants. Outcomes were incident diagnosis of coronary heart disease, heart failure (HF), chronic kidney disease, oedema and switching antihypertensive medication.

**Results:**

Participants were aged 40–79 years at first dCCB prescription. Carriers of rs877087 T allele in *RYR3* had increased risk of hazard ratio (HF 1.13: 95% confidence interval 1.02 to 1.25, *P* = .02). Although nonsignificant after multiple testing correction, the association is consistent with prior evidence. We estimated that if rs877087 T allele could experience the same treatment effect as noncarriers, the incidence of HF in patients prescribed dCCB would reduce by 9.2% (95% confidence interval 3.1 to 15.4). In patients with a history of heart disease prior to dCCB (*n =* 2296), rs877087 homozygotes had increased risk of new coronary heart disease or HF compared to CC variant. rs10898815 in *NUMA1* and rs776746 in *CYP3A5* increased likelihood of switching to an alternative antihypertensive. The remaining variants were not strongly or consistently associated with studied outcomes.

**Conclusion:**

Patients with common genetic variants in *NUMA1*, *CYP3A5* and *RYR3* had increased adverse clinical outcomes. Work is needed to establish whether outcomes of dCCB prescribing could be improved by prior knowledge of pharmacogenetics variants supported by clinical evidence of association with adverse events.

**What is already known about this subject**

- Antihypertensives are amongst the most commonly prescribed medications. The pharmacogenomics knowledge base PharmGKB documents genetic variants reported to influence antihypertensives effectiveness or adverse events. The levels of supporting evidence are variable and evidence of impact on clinical outcomes, especially in routine primary care (rather than in acute hospital care settings) is limited.

**What this study adds**

- We estimated the extent to which 23 commonly occurring pharmacogenetic variants reported to affect calcium‐channel blockers effectiveness are associated with clinical outcomes in the UK Biobank community cohort.
- We used a novel pharmacogenetic causal inference approach to estimate of the outcome if all participants had the low‐risk genotype. We found that if carriers of *RYR3* variant rs877087 could experience the same treatment effect as noncarriers the incidence of heart failure in patients prescribed calcium channel blockers would reduce by 9.2%.

## 1 INTRODUCTION

High blood pressure—hypertension—is a key modifiable risk factor for cardiovascular morbidity and mortality. While reducing raised blood pressures is the goal, only 1/3 of hypertensive patients treated with antihypertensive medications are estimated to reach target blood pressures.^1^ The reasons for failure to control raised blood pressure are complex, but genetic factors are proposed to play a role, either directly on blood pressure or indirectly by influencing antihypertensive medication response, adverse events or medication adherence.^2^

Calcium‐channel blockers (CCBs) are the first line recommended antihypertensive for most adults with hypertension, and their use is widespread across the world.^3^, ^4^, ^5^ There are 2 subgroups of CCBs, the most common being dihydropyridines (dCCBs), which are regarded as relatively safe and cost‐effective.^6^ Oedema is a common dCCB adverse effect, with incidence rates of 22%,^7^, ^8^, ^9^ that affects the quality of life of patients and can lead to discontinuation of treatment.^10^, ^11^ The presence of oedema can result in additional prescribing, which in turn can cause additional adverse outcomes including falls, over diuresis, acute kidney injury and polypharmacy.^11^, ^12^ Genetic factors can predispose to side effects, as well as further complications.

The pharmacogenomics knowledge base (PharmGKB) documents genetic variants reported to influence dCCB effectiveness or adverse events.^2^, ^13^, ^14^, ^15^ The levels of supporting evidence for each variant is variable, with many only having limited clinical evidence. Such evidence includes reported genes containing single‐nucleotide polymorphisms (SNPs) include those encoding calcium channel subunits themselves, such as the voltage‐gated calcium channels α1C (*CACNA1C*). SNPs in other ion channels are reported to alter dCCB responses (including *PICALM*, *TANC2*, *NUMA1*, *APCDD1*, *GNB3*, *SLC14A2*, *ADRA1A* , *ADRB2* and *CYP3A4*). SNPs in ATP‐binding cassette subfamily B member 1 (*ABCB1*) and in cytochrome p450 3A5 (*CYP3A5*) are reported to affect the clearance of dCCBs, and SNPs in cytochrome p450 ox reductase (*POR*) reportedly influence the plasma concentration of medicines. SNPs in nitric oxide synthase 1 adaptor protein (*NOS1AP*) increase risk of cardiovascular death, and SNPs in ryanodine receptor 3 ( *RYR3* )^16^ and in atrial natriuretic precursor A (*NPPA*) are reported to increase the risk of cardiovascular disease. In particular, *RYR3* (in intracellular calcium channels) was found to be associated with heart failure (HF) and there is a need to examine its effect on stroke, and effects on heart disease in risky groups as it is unknown.^17^

Evidence of impact on clinical outcomes, especially in routine primary care (rather than in acute hospital care settings) is currently limited for most pharmacogenetics variants reported to affect dCCBs. Here we analyse the UK Biobank (UKB) community volunteer cohort with linked genetic and medical records. We aimed to determine the extent to which 23 commonly occurring (minor allele frequency >3%) pharmacogenetic variants in 16 genes reported to affect dCCB effectiveness or rates of adverse events are associated with clinical outcomes.

## 2 METHODS

### 2.1 UKB cohort

The UKB enrolled 503 325 community‐based volunteers aged 40–70 years who visited 1 of 22 assessment centres in Wales, Scotland or England in 2006–2010.^18^ Extensive questionnaires on demographic, lifestyle and health information data were collected at the baseline assessment. Blood samples for genetic and biochemical analyses, and anthropometric measures were gathered. This study of dihydropyridines was conducted using the linked GP (primary care) data available in 230 096 participants. Data were available between January 1990 and August 2017 (see below for details). Participants gave consent to receive relevant information about clinical findings at baseline only: therefore, UKB data on individual genetic status were not reported to participants or their clinicians and could not therefore have influenced prescribing.

### 2.2 General practice data

More than 57 million prescriptions for 230 096 (45.7%) participants in the primary care data were recorded. The GP data were available up to 31 May 2016 (England TPP system supplier) and 31 August 2017 (Wales EMIS/Vision system). Drug name, quantity, date of prescription and drug code (in clinical Read v2, British National Formulary [BNF] or dm + d [Dictionary of Medicines and Devices] format, depending on suppler) are available. We used the UK National Institute for Health and Care Excellence (NICE) BNF database (https://bnf.nice.org.uk) to identify medication drug and brand names prescribed in the NHS that matched our search criteria for antihypertensives. Where another study included specific medications/brands we also include these. We included participants prescribed dihydropyridines, AND hence identified prescribing records for these medications (see Supporting Information for details), including date of each prescription.

We also identified antihypertensive prescriptions apart from dCCB; diuretics, β‐blockers, α‐blockers, angiotensin converting enzyme inhibitors and other antihypertensives using the Read 2 codes and BNF codes (see Table S1 for details).

We defined the censoring date for GP prescribing as either the date of deduction (removal from GP list, where available) or 31 May 2016 where no deduction date was present (i.e., still registered at an available practice). Data after 31 May 2016 are incomplete, depending on GP provider (see UKB documentation^19^).

### 2.3 Disease ascertainment

Primary and secondary care health records were used to examine the dCCB‐related adverse events. Peripheral oedema diagnoses were ascertained from ICD‐10 and ICD‐9 codes: and converted to Read codes used in UK primary care records using UKB‐provided diagnostic code maps. Cardiovascular events from hospital admissions records were available up to 14 years follow‐up after baseline assessment (HES in England up to 30 September 2020: data from Scotland and Wales censored to 31 August 2020 and 28 February 2018, respectively), covering the entire period up to the date of censoring of primary care prescribing data. Diagnosis of myocardial infarction (MI)/angina, stroke, chronic kidney disease (CKD), HF and ischemic stroke were ascertained using ICD‐10 codes (see Supporting Information for further details).

### 2.4 Genetic variants

We utilized genotype data from UKB, as described previously^20^ (see Supporting Information for details). Our analysis included 451 367 participants (93%) identified as genetically European (identified by genetic clustering, as described previously^21^): unfortunately, sample sizes from other ancestry groups were too small to analyse separately.

We analysed the genetic variants with documented effects on dCCBs effectiveness in the literature^22^ and in the PharmGKB database (March 2022). This included 29 SNPs in the following genes: *NPPA*, *NOS1AP*, *CYP3A4*, *GNB3*, *RYR3*, *CACNA1C*, *ABCB1*, *ADRA1A*, *SLC14A2*, *ADRB2*, *POR*, *PICALM*, *TANC2*, *NUMA1* and *APCDD1* (see Table S1 for details). Genotype status for 23 variants could be ascertained from the available UKB imputed data (release version 3) and minor allele frequencies were common enough to study (frequency varying from 3 to 46%—see Table S2 for details) in the UKB cohort: results for all 23 studied SNPs are reported. We calculated correlation coefficient to check linkage disequilibrium.

### 2.5 Primary analysis

Associations between genotypes and outcomes (GP‐diagnosed oedema and hospital‐diagnosed coronary heart disease [CHD; MI/angina], HF and CKD) were estimated using Cox proportional hazards regression models. See Supporting Information for further details of model specifics.

To estimate the genetically moderated treatment effect (GMTE), we used TWIST (Triangulation with a Study),^23^ a novel pharmacogenetic causal inference approach. This enables estimation of the predicted outcome if all participants were reassigned the low‐risk genotype, therefore providing an estimate of the genetic effect. In brief, the methods uses Aalen additive hazards regression models to test several assumptions common to pharmacogenetic analysis; primarily that the genetic variants do not predict whether an individual receives dCCB treatment; are not associated with any measured confounders predicting dCCB use or the studied outcome; and only affect the outcome through the interaction with dCCBs (see Bowden *et al*.^23^ for details). From this analysis, the most efficient and robust estimate of the GMTE is derived. Of note, the GMTE estimate may be the result of applying a single method, or instead be the combination of 2 or more estimates from different methods. The TWIST framework explicitly tests the association between genotype and outcome in the treated and untreated groups separately, to determine the GMTE independent of any effect in untreated individuals. We used R version 4.0.2 and R package *twistR* (https://github.com/lukepilling/twistR) v.0.1.3.

We also investigated the association between genotype and likelihood of switching dCCB for an alternative antihypertensive prescription using Cox's proportional hazards regression models, with adjustment for age at first prescription, sex and genotyping principal components of ancestry 1–10 in patients. See Supporting Information for further details.

To adjust for multiple statistical testing and control the false discovery rate, we applied Benjamini–Hochberg correction to *P* values for the associations between 23 SNPs and each outcome (using R function *p.adjust()*).

### 2.6 Secondary analysis in patients with heart disease diagnosis prior to dCCB treatment

We only included patients who had any heart diseases prior to the dCCB treatment for MI/angina/HF outcomes models as secondary analysis, as worsening angina and acute MI are reported as a caution for patients with coronary artery disease by the Food and Drug Administration in the prescribing information.^9^ We also tested associations for stroke and the *RYR3* calcium channel gene variant in patients on dCCBs, to examine the treatment effect, as stroke was associated with RYR3 in a genome‐wide association study (GWAS) regardless of use of dCCBs.^24^

### 2.7 Nomenclature of targets and ligands

Key protein targets and ligands in this article are hyperlinked to corresponding entries in http://www.guidetopharmacology.org and are permanently archived in the Concise Guide to PHARMACOLOGY 2019/20 (Alexander *et al*., 2019a,b).

## 3 SENSITIVITY ANALYSIS

### 3.1 Prescribed additional antihypertensives

We identified the BNF and Read 2 codes for the antihypertensive medication classes (β‐blockers, α‐blockers, diuretics, angiotensin receptor blockers, angiotensin converting enzyme inhibitors and vasodilators) and determined whether patients receiving dCCB prescriptions also received another antihypertensive within the dCCB prescribing time period. We then included this variable as a covariate in analyses.

### 3.2 Amlodipine and other dCCBs

We performed sensitivity analysis of our primary results splitting the dCCBs into 2 categories: in just those patients prescribed amlodipine (by far the most common dCCB) and other dCCB only and repeated the analyses described in the previous sections. Further splitting of nonamlodipine dCCBs was not feasible due to low numbers.

### 3.3 Analysis of unrelated participants only

We identified participants related to the third degree or closer using KING kinship analysis.^25^ We then repeated our primary results only in unrelated participants of European descent by randomly excluding 1 of each pair of related to the third degree or closer.

## 4 RESULTS

### 4.1 Characteristics of the sample

There were 32 360 (45.6% female) patients who were prescribed dCCB in primary care. The mean age was 61.3 years (standard deviation [SD] 7.7). The number of prescriptions in a year varied from 1 to 25, with a mean of 9.2 (SD 4.6) and a median of 7.9 (interquartile range 6.3 to 13). The mean prescription period was 5.9 (SD 5.2) years, the median was 4.4 (interquartile range 1.6 to 9.1) (see Table 1 for details). The allele frequencies for the 23 studied genetic variants range from 3 to 50% (for details, see Table S3). We found no pairs of variants in high (*R* ^2^ < .8) linkage disequilibrium (see Table S4).

**TABLE 1:** Descriptive table of the UK Biobank participants included in the analysis

GP prescribed	
Number of participants	32 360
Females, *n* (%)	17 590 (45.6)
Age at first prescription	
Range	40–79.3
Mean (SD)	61.3 (7.7)
Number of prescriptions in a year	
Range	1–25
Mean (SD)	9.2 (4.6)
Years between first and last prescription	
Range	0.08–39.9
Mean (SD)	5.9 (5.2)
Oedema^a^ preprescriptions of dCCBs	3470 (8.8)
MI/angina^b^ preprescription of dCCBs	3026 (7.7)
CKD^b^ preprescriptions of dCCBs	229 (0.6)
Heart failure^b^ preprescriptions of dCCBs	333 (0.8)
Stroke^b^ preprescription of dCCBs	333 (0.8)
Prescribed other antihypertensive	23 971 (61%)
Changing treatment from dCCBs to others	4127 (10.5)
Oedema^a^ postprescriptions of dCCBs	5913 (15.04)
Myocardial infarction/angina^b^ postprescription of dCCBs	7430 (18.9)
CKD^b^ postprescriptions of dCCBs	2940 (7.5)
Heart failure^b^ postprescriptions of dCCBs	2292 (5.8)
Stroke^b^ postprescription of dCCBs	1001 (2.6)

European‐ancestry participants with >1 dCCB prescription in the available GP prescribing data.

CKD, chronic kidney disease; dCCB, dihydropyridine calcium‐channel blocker.

^a^ Primary care recorded.

^b^ Hospital diagnosed diseased.

### 4.2 Associations with prior evidence

We investigated 23 genetic variants with reported pharmacogenetic effects on dCCB effectiveness or adverse events. We found supporting evidence in the UKB for 5 of the 23 reported dCCB pharmacogenetic associations (Table 2). Details of the 5 genes reported below, including secondary analysis of other adverse outcomes.

**TABLE 2:** Significant associations in our study and how they have found previously in the literature

Gene	rs ids	What is found?	What have we found?	Reference	Paper
NUMA1	rs10898815	G^a^ alleles carriers have better response to amlodipine	AA is associated with increased treatment switch	PharmGKB	^29^
ADRA1A	rs1048101	Genotypes AA + AG are associated with increased response compared to GG	AG and GG are associated with decreased CKD	PharmGKB	^36^
APCDD1	rs564991	C alleles carriers have better response to dCCB	C allele carriers have increased CHD and decreased CKD	Other	^29^
CYP3A5	rs776746	T allele carriers may have decreased metabolism of dCCB	TT is associated with increased CKD and treatment switch	PharmGKB	^30^
RYR3	rs877087	Genotype TT is associated with increased HF	T allele is associated with HF, in subgroup CT is associated with stroke and HF/MI/angina	PharmGKB	^17^

^a^ Paper reported C allele on the reverse strand whereas we have reported the alleles with respect to the forward strand.

CHD, coronary heart disease; CKD, chronic kidney disease; dCCB, dihydropyridine calcium‐channel blocker; HF, heart failure; MI, myocardial infarction

#### 4.2.1 RYR3

The *RYR3* rs877087 T allele prevalence in people on dCCB treatment in UKB was 46%, and TT homozygotes was 21.3%.

**FIGURE 1:**
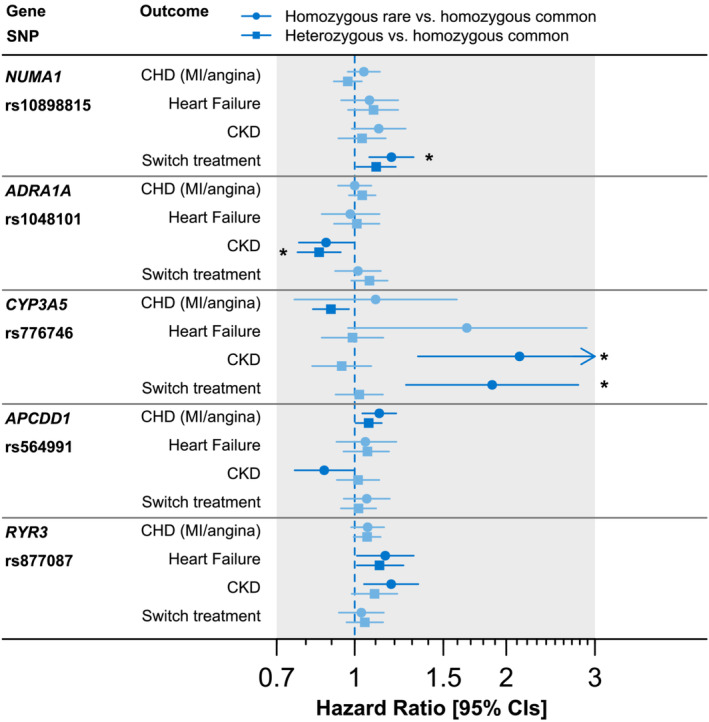
Associations between top 6 genotypes and dihydropyridine calcium‐channel blocker (dCCB)‐related adverse events

Of the 32 360 patients prescribed dCCBs, 2292 developed HF during the follow‐up period. Diagnoses were more common in *RYR3* rs877087 TT homozygotes (*n* = 404, 6.1% of 6607) and CT heterozygotes (*n* = 943, 6.1% of 15 377) compared to common CC homozygotes (*n* = 491, 5.4% of 9090; Figure 1 and Table 3; see Table S5 for details). The increased risk of hospital diagnosed HF was significant in Cox's proportional hazards regression (HR) models adjusted for age at first dCCB prescription, sex and genetic ancestry (HR_TT vs. CC_ 1.15, 95% confidence interval [CI] 1.01 to 1.31, *P =* .04 and HR_CT vs. CC_ 1.12, 95% CI 1.01 to 1.25, *P* = .04), and length of treatment is explicitly modelled in the time‐to‐event analysis methods. We also performed an analysis of rs877087 assuming a dominant model of inheritance, given the similarity in estimates between the CT and TT groups: T‐allele carriers had 13% increased risks of HF compared to CC homozygotes (HR 1.13: 95% CI 1.02 to 1.25, *P* = .02). These results were not significant after Benjamini–Hochberg adjustment for multiple statistical testing (adjusted *P* > .05). Heterozygotes (11.5%) were also more likely to have incident MI, angina or HF compared to common CC homozygotes (10.3%; HR 1.12, 95% CI 1.03 to 1.21, *P* = .007; Table 4). Heterozygotes were more likely to have incident stroke compared to CC homozygotes (HR 1.22, 95% CI 1.04 to 1.45, *P* = .02; Table 3).

**TABLE 3:** Significant associations between genotypes and dihydropyridine calcium‐channel blocker‐related adverse events

Outcome	Gene	Allele	*N*	*n*	%	HR	95% CI		*P*
Switching treatment	*NUMA1*	GG	8463	700	8.27				
	rs10898815	GA	16 632	1506	9.05	1.10	1.01	1.21	.03
		AA	8272	800	9.67	1.18	1.07	1.31	.00
			33 367	3006					
	*CYP3A5*	CC	29 126	2608	8.95				
	rs776746	CT	4135	376	9.09	1.02	0.92	1.14	.70
		TT	156	25	16.03	1.87	1.26	2.78	.00
			33 417	3009					
Chronic kidney disease	*CYP3A5*	CC	25 177	1757	7.0				
	rs776746	CT	3545	239	6.7	0.94	0.82	1.08	.39
		TT	140	18	12.9	2.12	1.34	3.38	.002
			28 862	2014					
	*RYR3*	CC	8467	545	6.4				
	rs877087	TC	14 231	1008	7.1	1.10	0.99	1.22	.09
		TT	6164	461	7.5	1.18	1.04	1.34	.01
			28 862	2014					
	*APCDD1*	AA	9709	675	7.0				
	rs564991	AC	14 179	1027	7.2	1.02	0.92	1.12	.76
		CC	4910	306	6.2	0.87	0.76	1.00	.04
			28 798	2008					
	*ADRA1A*	AA	8751	672	7.7				
	rs1048101	AG	14 277	947	6.6	0.85	0.77	0.94	.001
		GG	5834	395	6.8	0.88	0.77	0.99	.04
			28 862	2014					
Heart failure	*RYR3*	CC	9090	491	5.4				
	rs877087	CT	15 377	943	6.1	1.12	1.01	1.25	.040
		TT	6607	404	6.1	1.15	1.01	1.31	.04
			31 074	1838					
Coronary heart disease	*APCDD1*	AA	10 299	1770	17.2				
	rs564991	AC	15 111	2771	18.3	1.07	1.00	1.13	.04
		CC	5262	1004	19.1	1.12	1.04	1.21	.004
			30 672	5545					

*N*, total number of genotype group in analysis; *n*, number of diagnoses; %, percent of genotype group with a diagnosis.

**TABLE 4:** Associations between hospital‐diagnosed coronary heart disease (CHD) and stroke and variants of *RYR3* in patients with CHD diagnosed prior to dihydropyridine calcium‐channel blocker prescription

Outcome	Genotype	*n*	*N*	%	HR	0.95	CI	*P*
Stroke	CC	203	9091	2.2				
	CT	426	15 381	2.8	1.22	1.04	1.45	.02
	TT	164	6609	2.5	1.12	0.91	1.38	.27
HF or MI or angina	CC	926	8981	10.3				
	CT	1746	15 206	11.5	1.12	1.03	1.21	.01
	TT	722	6545	11	1.1	1	1.21	.06
HF or MI or angina in high‐risk group ^a^	CC	433	639	67.8				
	CT	825	1185	69.6	1.1	0.98	1.23	.12
	TT	356	472	75.4	1.25	1.09	1.44	.002

^a^ We examined genotype effect on CHD in patients with a CHD history prior to the dihydropyridine calcium‐channel blocker prescriptions.

HF, heart failure; MI, myocardial infarction

We used the TWIST^23^ framework to estimate that the overall incidence of HF in patients prescribed dCCBs could be reduced by 9.2% (95% CI 3.1 to 15.4) if rs877087 T allele carriers received the same treatment benefit as noncarriers, that is, were switched to an alternative antihypertensive medication unaffected by rs877087 genotype.

To give further details on the TWIST results: because the association with HF was similar between heterozygotes and minor allele homozygotes we estimated the GMTE in carriers (any rs877087 T allele) compared to CC homozygotes. rs877087 was not associated with HF in individuals never prescribed dCCBs (GMTE0 estimate *P* > .05; Table S6). From TWIST we found the *robust* GMTE and the *Mendelian randomization* estimates could be combined to give a more efficient and precise estimate. The risk of HF was 0.069% greater per year after treatment initiation in carriers compared to noncarriers (*P* = .003; Table S6). When multiplied by the number of genotype‐carrier patient‐years in the model (244 818) and divided by the total number of diagnoses in the treated individual (1838), we estimate that if carriers of the rs877087 T allele could experience the same treatment effect as noncarriers, 170 HF diagnoses could have been avoided (95% CI 58 to 282), hence the 9.2% quoted earlier.

In the subgroup of patients with a pre‐existing heart disease (MI, angina or HF) at the start of dCCB prescribing, *RYR3* TT homozygotes had an increased risk of developing incident heart diseases compared to common CC homozygotes (75.4 *vs*. 67.8) with a HR 1.25 (95% CI 1.09 to 1.44, *P* = .002; Table 3; see Table S7).

Overall, 2940 (7.5%) patients on dCCBs had incident CKD. *RYR3* rs877087 TT homozygotes (461 CKD cases in 6164 TT homozygotes) were more likely to have hospital‐diagnosed CKD compared to the common homozygotes groups (HR 1.18, 95% CI 1.04 to 1.34, *P* = .01; see Table S8 for details). We estimate that if carriers of the rs877087 T allele could experience the same treatment effect as noncarriers, 199 CKD diagnoses could have been avoided (95% CI 75 to 324; see Table S6). Therefore, the overall incidence of CKD in patients prescribed dCCBs could be reduced by 8.6% if rs877087 T allele carriers received the same treatment benefit as noncarriers (95% CI 3.2 to 14.0).

#### 4.2.2 CYP3A5

Patients with *CYP3A5* rs776746 TT (CYP3A5*3) genotype (0.47% of patients), a variant previously linked to kidney related outcomes^26^ had increased risk of CKD (HR 2.12: 95% CI 1.34 to 3.38, *P* = .002) compared to CC homozygotes (Figure 1 and Table 3). The association was still significant after Benjamini–Hochberg adjustment for multiple statistical testing (adjusted *P* = .03). When we repeated the analysis for patients who were on dCCB but had no CKD history, 12.3% of *CYP3A5* rs776746 TT homozygotes without prevalent CKD were diagnosed with incident CKD compared to 6.6% of heterozygotes and 6.8% homozygotes for CC (HR 2.09, 95% CI 1.29 to 3.37, *P* = .003; see Table S9 for details). We estimated that if rs776746 TT homozygotes could experience the same treatment effect as CC homozygotes 11 CKD diagnoses could have been avoided (95% CI 4 to 18; see Table S6). Therefore, the overall incidence of CKD in patients prescribed dCCBs could be reduced by 0.5% (95% CI 0.2 to 0.9) if rs776746 TT homozygotes received the same treatment benefit as CC homozygotes.

Of the patients on dCCB prescription, 5565 (14.2%) changed treatment from dCCB CCBs to other antihypertensives. *CYP3A5* rs776746 TT homozygotes (*n* = 27/152) were also more likely to change treatments compared to common homozygotes; HR 1.59, 95% CI 1.09 to 2.32, *P* = .02, respectively (see Figure 1 and Table 3; see Table S10 for details). Incident MI/angina was less likely to occur in patients heterozygous for *CYP3A5* rs776746 compared to CC homozygotes (*P* = .01).

#### 4.2.3 NUMA1

Of those 3006 patients switched treatment, 800 were *NUMA1* rs10898815 AA homozygotes (*n* = 8272), and 1506 were GA heterozygotes (*n* = 16 632). AA homozygotes and GA heterozygotes were more likely to switch treatments compare to their common homozygotes (HR 1.18, 95% CI 1.07 to 1.31, *P =* .001 and HR 1.10, 95% CI 1.01 to 1.21, *P* = .03, respectively; Figure 1 and Table 3; see Table S10 for details). The association for AA was still significant after Benjamini–Hochberg adjustment for multiple statistical testing (adjusted *P* = .04).

#### 4.2.4 ADRA1A

Adrenoceptor α1A (*ADRA1A*) rs1048101 AA homozygotes had an increased risk for CKD (HR 1.18, 95% CI 1.04 to 1.34, *P* = .01) compared to GG homozygotes. GG homozygotes (395 cases of 5834 patients) and AG heterozygotes (947 cases of 14 277 patients) were associated with decreased risk of CKD compared to AA homozygotes (HR 0.88, 95% CI 0.77 to 0.99, *P* = .04, false discovery rate [FDR] *P* = .25 and HR 0.85, 95% CI 0.77 to 0.94, *P* = .001, FDR *P* = .03, respectively; Figure 1 and Table 3). We estimated that if rs1048101 AA homozygotes could experience the same treatment effect as noncarriers (e.g., were prescribed an alternative antihypertensive medication unaffected by this genotype) 86 CKD diagnoses could have been avoided (95% CI 13 to 138; see Table S6). Therefore, the overall incidence of CKD in patients prescribed dCCBs could be reduced by 7% (95% CI 1.1 to 12.9) if rs1048101 AA homozygotes received the same treatment benefit as GG homozygotes.

#### 4.2.5 APCDD1

Of those patients on dCCB prescriptions, 7430 (18.9%) patients had incident MI/angina post‐dCCB treatment. Of those 7430 patients, 1004 were homozygotes for *APCDD1* rs564991 CC; 19.1% homozygotes for *APCDD1* rs564991 CC had increased risk for MI/angina compared to 18.3% heterozygotes and 17.2% homozygotes for AA (HR 1.12, 95% CI 1.04 to 1.21, *P =* .004, FDR *P* = .2 for CC and HR 1.07, 95% CI 1 to 1.13, *P* = .04, FDR *P* = .28 for AC; Figure 1 and Table 3; see Table S11 for details). TWIST analysis showed that rs564991 was not associated with CHD in individuals never prescribed dCCBs (GMTE0 estimate *P* > .05; Table S4). The risk of CHD was 0.19% (*P* = .002) greater per year after treatment in CC homozygotes compared to AA homozygotes (Table S6). We estimated that if rs564991 CC homozygotes could experience the same treatment effect as AA homozygotes 98 CHD diagnoses could have been avoided (95% CI 35 to 162). Therefore, the overall incidence of CHD in patients prescribed dCCBs could be reduced by 3.5% (1.3 to 5.8) if rs564991 CC homozygotes received the same treatment benefit as AA homozygotes.

Of 4910 *APCDD1* rs564991 CC homozygotes, 306 patients had CKD after dCCB treatment. They were less likely to have CKD compared to their common homozygotes (6.2% *vs*. 7%; HR 0.87, 95% CI 0.76 to 1, *P* = .04). However, these were not significant after adjusting for multiple statistical testing. TWIST analysis estimated that is rs564991 CC homozygotes experienced the same treatment effect as AA homozygotes (i.e., were switched to an alternative antihypertensive) this may increase the overall CKD incidence in patients prescribed dCCBs by 63 diagnoses (95% CI 23 to 104, *P* = .002).

GP‐diagnosed post‐dCCB oedema (*n* = 5913—15.04%) was not associated with any of the variants. See Table S12 for details.

See Supporting Information for summary of associations for variants in other genes.

## 5 SENSITIVITY ANALYSES

In total, 23 971 (61%) patients were also on another antihypertensive medication at some point during dCCB prescription time period. In the sensitivity analysis adjusted for receiving another antihypertensives during the dCCB prescribing period, significant associations with outcomes from the main analysis remained consistent. See Table S13 for the details.

The sensitivity analysis of patients on amlodipine (*n* = 31 357) and other dCCBs (*n* = 6854) separately were consisted with the primary analysis, is presented in Table S14.

After excluding one of patients related 27 042 patients remained. Many associations remained significant such as *APCDD1* and CHD, *RYR3* and CKD, *NUMA1* and treatment switch whilst some associations were not significant, the effect sizes were consistent to the whole cohort (see Table S15).

## 6 DISCUSSION

CCBs, especially dihydropyridines (dCCBs) such as amlodipine, are commonly prescribed to reduce blood pressure. Many pharmacogenetic variants are reported to impact dCCB responses, with evidence from laboratory studies, randomized trials, or acute hospital settings. However, data on clinical impact in routine care in the community is limited. We estimated the association between 23 pharmacogenetic variants reported to affect dCCB response or adverse events in 32 360 patients prescribed dCCB using the UKB‐linked primary care data. Outcomes were assessed over a mean follow up of >10 years after first dCCB prescription. The most striking results were for the ryanodine receptor 3 (*RYR3*) rs877087, with T allele carriers having 13% increased risks of HF (*P* = .02), after accounting for any effect in untreated individuals. Although results were not significant after Benjamini–Hochberg adjustment for multiple statistical testing (adjusted *P* > .05), the burden of prior evidence increases plausibility of the associations. In patients with a history of heart disease when first prescribed dCCB (*n =* 2296), *RYR3* homozygotes had 25% increased risk of heart disease diagnosis (MI, angina or HF) compared to CC homozygotes (*P =* .002). In addition, 2 genetic variants increased the likelihood of patients switching to an alternative antihypertensive medication (in *NUMA1* and *CYP3A5*). The variant in *CYP3A5* also increased risk of CKD, and we hypothesize that it might be the reason for the switch in treatment, whilst the variant *APCDD1* increased risk of CHD. These adverse reactions are potentially preventable if patients were prescribed medications accounting for genotype. However, for the majority of reported pharmacogenetic variants included, we found little or inconsistent evidence of associations with adverse events, and some appeared genetically contradictory (with heterozygote and homozygote effects in opposite directions). Many associations had modest *P* values that would not survive strict multiple testing corrections.

*RYR3* mediates Ca^2+^ release from ryanodine‐sensitive stores, triggering cardiac and skeletal muscles.^27^ A common variant in *RYR3* (rs877087) increased risk of HF in a study of 2516 people randomized to amlodipine or to other antihypertensives.^17^ We support and extend this literature in a substantially larger sample using longitudinal analysis methods: we report increased HF risk in both TT homozygotes (*n* HFs = 404 in 6607 genotypes; HR 1.15) and CT heterozygotes (*n* HFs = 943 in 15 377 genotypes; HR 1.12), compared to CC homozygotes (*n* = 9090). We used TWIST,^23^ a novel pharmacogenetic causal inference framework, to estimate the population average GMTE on HF if all *RYR3* T allele carriers could experience the same treatment effect as common CC homozygotes (e.g., they were prescribed an alternative medication): we estimate that HF risk would reduce by 9.2%, corresponding to 170 avoidable HF diagnoses in the studied patients. Further work is needed to determine the optimum strategy to reduce the risk of T allele carriers, for example, by prescribing an alternative treatment or with increased monitoring of patients. Furthermore, rs877087 has been associated with stroke in a GWAS, but the genotype effect in patients on treatment is unknown.^24^ Our findings suggest that rs877087 CT had an increased risk of hospital diagnosed stroke (*n* = 203/15381, HR = 1.11, *P* = .02) compared to common CC homozygotes, but we found effect in TT homozygotes.

Observational studies of drug effects often suffer from indication and other biases: As doctors aim to prescribe each medication based on the patients' clinical state, statistically separating the effects of the medication from the effects of underlying disease is challenging, especially as data to correct potential confounders are seldom complete or entirely accurate. However, genotypes are inherited at conception and stay fixed, meaning that they predate receipt of the studied medications. In our study, we found that genotype was not associated with treatment initiation. Associations between genotypes and outcomes provide less confounded evidence than conventional observational associations, particularly because the participants and GPs were not told given genotype information by the UKB study. Because genetic variants are largely independent of traditional confounding,^28^ and GPs and patients are unaware of these genotypes when making prescribing decisions and diagnosing outcomes, we can therefore assume that the difference between genotype carriers is due to the modifying effect of the genotype on medication (hence our naming in the GMTE ‐genetically modified treatment effect). This assumption is common to such Mendelian randomization studies. Therefore, our finding that variants in 2 genes (*NUMA1* and *CYP3A5*) with switching treatment may result directly from dCCB pharmacokinetics or/and pharmacodynamics effects. Further work (both replication and experimental validation) is required to confirm the precise biological mechanisms involved.

*NUMA1* rs10898815 was previously identified in a GWAS of blood pressure^29^ but we found no reports on switching antihypertensive treatment. We found that AA homozygotes had increased likelihood of switching treatment, which was significant after multiple testing adjustment. CYP3A5 is a cytochrome p450 enzyme and metabolizer of dCCB; CYP3A5*3 is the most common nonfunctional allele (rs776746‐T; with a prevalence of 6.6% in the UKB European cohort), which results in increased clearance of dCCB,^30^ resulting in less successful treatment. Our findings support this: CYP3A5*3 homozygotes had increased risk of CKD, for which high blood pressure is a risk factor, and were 59% more likely to change treatments compared to common homozygotes.

In a pathway‐focused GWAS,^31^ genes in the *ADRA1* pathway ultimately affect intracellular calcium release (which dCCBs block) and blood pressure. The isoforms (e.g., *ADRA1A*) was associated with hypertension in patients. In a study in mice, it also mediated renal vasoconstriction in hypertension.^32^ Furthermore, *ADRA1A* affects renal functions via regulating Na^+^ reabsorption, renin secretion, renal blood flow and glomerular filtration rate, of which alterations cause kidney disease.^32^, ^33^ Although we were not able to analyse GP‐recorded blood pressure measures robustly due to high rates of missing data, an SNP in *ADRA1A* (rs1048101) was associated with CKD. Patients homozygous for the common AA allele (30.4% of participants) had an increased risk of CKD, in contrast to GG homozygotes (20.3%), who had decreased risk. In TWIST analysis, we estimated that 86 CKD diagnoses (7% of total) could be avoided if AA homozygotes could receive an alternative antihypertensive unaffected by the genotype.

In a previous GWAS, the rs564991 C allele in *APCDD1* was associated with response to CCB.^29^ In our study, we found that CC homozygotes had increased risk of MI/angina with a HR 1.12 (*P* = .004); however, this was not significant after accounting for multiple testing.

It is important to consider concomitant medications to fully interpret our results. To evaluate whether coprescribing with other antihypertensive medications during patients' dCCB prescribing period affected the results, we conducted a sensitivity analysis adjusting for whether the patient received another antihypertensive. The significant associations we report in the primary analysis were consistent and remained significant. We opted to adjust rather than exclude, as excluding could introduce bias as extra medications might be prescribed for the adverse events (i.e., oedema) or worsening conditions (i.e., HF) we aimed to study and also reduce power. To determine whether results are biased by including multiple dCCBs in the analysis, which may have divergent mechanisms despite being in the same drug class, we performed sensitivity analysis. Subsetting the analysis into just amlodipine prescriptions and *other dCCB* demonstrated consistent effect sizes between the variants and outcomes, albeit with attenuated significance (due to reduced sample size), suggest that the significant associations we observe were not driven by a single dCCB drug.

In our analyses, 15.04% of patients on dCCBs had GP recorded oedema, lower than the reported prevalence of approximately 22% in the literature.^7^, ^8^ This could be due to limitations in the data available; UKB‐linked primary care diagnoses include diagnostic codes only, with no free text. It has been discussed previously that estimates of the prevalence of oedema depends on the study methods; in randomized controlled trials self‐reported oedema might be overestimated by the patients, or milder forms of oedema might be reported compared to those that enter the GP record.^8^

In routine clinical care, patients' blood pressure is regularly monitored after dCCB treatment initiation to determine whether the targets were achieved. However, we were unable to analyse this using UKB‐linked primary care data due to the sparsity of blood pressure data available (only a feew patients had blood pressure in the record at the time of initiation, or within 2 months). This wide variation between patients in the time from prescription to next follow up meant that we focused instead on adverse drug reactions, not measured blood pressure.

The PharmGKB database (*n* = 19 of the 23 studied variants were reported in PharmGKB) includes smaller and candidate studies, with only low or moderate levels of evidence for most of the relevant variants (as categorized by PharmGKB curators, often reflecting small sample sizes and lack of replication). Previously, the most frequently studied genes were *CYP3A4* and *CYP3A5* with smaller sample sizes or non‐European ancestry patients. The biggest study that we are aware of was a randomized control study 8174 patients randomized to amlodipine.^34^ Additionally, a previous review about pharmacogenomics of hypertension medications reported 4 variants associated with dCCB response in a small Japanese sample.^22^ Of the 23 dCCB variants, we found evidence for an effect on outcomes/adverse events for only 10 variants—even fewer after adjustment for multiple statistical testing—suggesting that these few specific variants should be a priority for future study. The possible reasons for the lack of consistency include interethnic differences in studied populations, heterogeneity in exact phenotype studied, lack of adherence to medication or variability in medication history between patients, but may also include publication biases, in which false positive statistical associations (type 1 errors) tend to be overrepresented, especially from small studies. However, we here add substantially to the evidence base for these variants due in part to the large sample size studied, but also the strengths of analysing real‐world primary care prescribing and the novel pharmacogenetic analysis approach triangulating evidence from multiple analysis methods (TWIST^35^). Using these data for pharmacogenetics analysis means that we are able to look at more adverse reactions over longer periods and have therefore increased confidence for the relevance to routine clinical care of hypertension of variants where significant effects on outcomes are identified.

In conclusion, our analysis of longer‐term prescribing in real‐world primary care data support the hypothesis that use of genetic information in antihypertensive prescribing might optimize treatment selection for specific patients to maximize efficacy and reduce incidence of adverse events. The variants identified as associated with adverse clinical outcomes are good candidates for studies to test whether dCCB treatment outcomes can be improved with pharmacogenetic guided prescribing.

## COMPETING INTERESTS

All authors declare no support from any organization for the submitted work, no financial relationships with any organizations that might have an interest in the submitted work in the previous 3 years and no other relationships or activities that could appear to have influenced the submitted work.

## CONTRIBUTORS

D.T. generated data, performed analyses, interpreted results, created the figures, searched the literature and cowrote the manuscript. J.A.H.M. provided expert clinical interpretation of the data and contributed to the manuscript. C.‐L.K., J.D. and J.B. contributed to data analysis and interpretation and contributed to the manuscript. D.M. oversaw interpretation and literature searching and cowrote the manuscript. L.C.P. generated data, performed analyses, interpreted results, created the figures, searched the literature and cowrote the manuscript.

## TRANSPARENCY

We affirm that this manuscript is an honest, accurate and transparent account of the study being reported; that no important aspects of the study have been omitted; and that any discrepancies from the study as planned (and, if relevant, registered) have been explained. The full methods are available in the Supporting Information.

## Supporting information


**Data S1.** Supporting information

**Data S2.** Supporting information

## Data Availability

The genetic and phenotypic UKB data are available upon application to the UKB (www.ukbiobank.ac.uk/register-apply). The derived data fields used in our analysis will be available via the UKB, searching for application number 14631—we are not able to share these directly.
