# Supplementary material for: Calcium‐channel blockers: Clinical outcome associations with reported pharmacogenetics variants in 32 000 patients
Source: Br J Clin Pharmacol. 2022 Oct 6;89(2):853–64. doi: 10.1111/bcp.15541 (PMC10091789; doi:10.1111/bcp.15541)
Supplement: Supplementary file 2 — Data S2. Supporting information [file BCP-89-853-s001.docx]

**Calcium Channel Blockers: clinical outcome associations with reported pharmacogenetics variants in 32,000 patients**

Türkmen et al.

**Supplementary Information**

[Supplementary Methods 1](#_Toc112229650)

[List of antihypertensive medications identified, including brand names 1](#_Toc112229651)

[Disease ascertainment 1](#_Toc112229652)

[Genotyping of UK Biobank 2](#_Toc112229653)

[Associations between genotypes and adverse events 2](#_Toc112229654)

[Associations between genotypes and switching treatment 2](#_Toc112229655)

[Supplementary Results 3](#_Toc112229656)

[Additional associations between variants in other genes and adverse events 3](#_Toc112229657)

[References 3](#_Toc112229658)

# Supplementary Methods

## List of antihypertensive medications identified, including brand names

Amlodipine: Norvasc®, Katerzia®, Istin®, Amlodipine®; felodipine: Plendil®, Cardioplen®, Folpik®, Felotens®, Vascalpha®, Neofel®, Parmid®; lacidipine: Lacipil®, Motens®; Molap®, Lacidipine®; nisoldipine: Sular®, lercanidipine: Zanidip®, Lercanidipine®, nimodipine: Nimotop®, Nymalize®, nifedipine: Adalat®, Afeditab®, Procardia®, Adipine®, Nifedipress®, Tensipine®, Dexipress®, Valni®, Adanif®, Neozipine®, Nidef®, Nifedipine®, Fortipine®, Coracten® ; nitrendipine: Baypress®, nicardipine: Cardene®.

## Disease ascertainment

Primary and secondary care health records were used to examine the dCCB related adverse events. Edema diagnosis was ascertained from ICD-10 and ICD-9 codes(19) :we included ICD-10 codes (R60.0 Localized edema, R60.1 Generalized edema, R60.9 edema, unspecified) and ICD-9 codes (7570, 7823, 2766) for edema. andWe converted these to Read codes (C3661, C3662, M2A.., R023., PH0.., PH00., PH01., PH02., PH0z., R023., C366.) used in UK primary care records using UK Biobank-provided diagnostic code maps.

Cardiovascular events from hospital admissions records were available up to 14 years follow-up after baseline assessment (HES in England up to 30 September 2020: data from Scotland and Wales censored to 31 August 2020 and 28 Feb 2018, respectively), covering the entire period up to the date of censoring of primary care prescribing data. Diagnosis of MI/angina was ascertained using ICD-10 codes I20*, I21*, I22*, I23*, I24* and I25*; ischemic stroke was using ICD-10 codes I63*; chronic kidney disease (CKD) using ICD10 codes N18* and Y84.1; and heart failure using ICD10 codes I50* and J81*.

## Genotyping of UK Biobank

Directly genotyped genetic variants (n=805,426) were ascertained in 488,377 UK Biobank participants, which used two almost identical platforms sharing >95% of variants: the Affymetrix Axiom UKB array (in 438,427 participants) and the Affymetrix UKBiLEVE array (in 49,950 participants). Extensive quality control was applied by the central UK Biobank team (1). Genotype imputation increased the number of genetic variants to ~96 million in 487,442 participants (1).

## Associations between genotypes and adverse events

These (GP-diagnosed edema, and hospital-diagnosed CHD (MI/angina), HF, and CKD) were estimated using Cox proportional hazards regression models. They were adjusted for age at first prescription, sex, and genetic principal components 1 to 10 (to adjust for population genetic ancestry) in patients who were prescribed dCCBs by their GPs (General Practice). We included patients if they have had at least two prescriptions and more than 2 months of dCCBs treatment. Patients exited the model on the hospital inpatient record censoring dates i.e., for cardiovascular and renal outcomes we therefore performed an ‘intention to treat’ analysis, reducing any bias due to discontinuation before diagnosis of adverse outcomes (e.g., discontinuing dCCB treatment and having heart diseases as a result). For the analysis of edema participants exited the model on the date of their last known prescription plus 2 months (the wash out period), as edema symptoms are alleviated after stopping medication.

For the analysis of edema diagnosis during dCCB prescribing we excluded patients with prior edema diagnoses. For the analysis of CKD diagnosis during dCCB prescribing we first performed the analysis in all patients, then repeated as secondary analysis excluding patients with CKD diagnosis prior to first dCCB prescription. For the analysis of acute MI and angina we did not exclude prevalent cases as worsening angina and acute MI are reported as a caution for patients with coronary arteria disease (2) in the prescribing information, and are therefore indications for dCCB prescribing.

Unless otherwise stated all analyses were performed in STATA v16.

## Associations between genotypes and switching treatment

Association between genotype and likelihood of switching dCCB for an alternative antihypertensive prescription were estimated using Cox proportional hazards regression models. We considered the deduction date provided i.e., if patients were removed from a GP practice included in the UK Biobank data this became their date of censoring. We included patients if they had 3 months of GP data after their last known prescription (i.e., we can be more certain of a discontinuation of dCCB treatment rather than the last prescription being too close to the date for censoring).

# Supplementary Results

## Additional associations between variants in other genes and adverse events

There were additional associations with specific outcomes for several other variants, although as these were based on very small numbers or were combined with contrary results between heterozygotes and homozygotes, these results should be treated with caution.

Patients heterozygotes for G protein subunit beta 3 (GNB3) rs5443 (n=2,434/13,017) had an increased risk for MI/angina compared to homozygotes for CC (HR 1.07: 95%CI 1.01 to 1.13, p=0.02). Incident MI/angina was less likely to occur in patients heterozygous for CYP3A4 rs2740574, and Nitric oxide synthase 1 adaptor protein (NOS1AP) rs12143842 (p=0.04, and p=0.03, respectively) compared to their common homozygotes.

P3A4 rs2740574 CC homozygotes (n=4/26) had an increased risk for hospital diagnosed CKD compared to their common homozygotes groups (HR 1.14: 95% CI 1.01 to 1.29, p=0.04 and HR 3.01: 95%CI 1.13 to 8.02, p=0.03, respectively). APCDD1 rs564991 CC homozygotes, NOS1AP rs10494366 heterozygotes (or GG+GT) and NOS1AP rs12143842 heterozygotes (or TT+TC) were less likely to have CKD compared to their common homozygotes.

# References

1. Bycroft C, Freeman C, Petkova D, Band G, Elliott LT, Sharp K, et al. The UK Biobank resource with deep phenotyping and genomic data. Nature. 2018;562(7726):203–9.

2. Food and Drug Administration. NORVASC® (amlodipine besylate) Prescribing Information. 1987;1–12. Available from: https://www.accessdata.fda.gov/drugsatfda_docs/label/2011/019787s047lbl.pdf
